# Musculoskeletal pain and its association with health status, maturity, and sports performance in adolescent sport school students: a 2-year follow-up

**DOI:** 10.1186/s13102-022-00437-x

**Published:** 2022-03-21

**Authors:** Julia S. Malmborg, Ann Bremander, Stefan Bergman, Emma Haglund, M. Charlotte Olsson

**Affiliations:** 1 Department of Environmental and Biosciences, School of Business, Innovation and Sustainability, Halmstad University, Box 823, 301 18 Halmstad, Sweden; 2 Spenshult Research and Development Center, FoU Spenshult, Bäckagårdsvägen 47, 302 74 Halmstad, Sweden; 3 Department of Regional Health Research, University of Southern Denmark, Winsløvsparken 19.3, 5000 Odense, Denmark; 4 Danish Hospital for Rheumatic Diseases, University Hospital of Southern Denmark, Engelshøjgade 9A, 6400 Sønderborg, Denmark; 5 Department of Clinical Sciences Lund, Section of Rheumatology, Lund University, Box 117, 221 00 Lund, Sweden; 6 Primary Health Care Unit, Department of Public Health and Community Medicine, Institute of Medicine, The Sahlgrenska Academy, University of Gothenburg, Box 454, 405 30 Gothenburg, Sweden

**Keywords:** Health status, Maturity, Sports performance, Epidemiology, Exercise physiology

## Abstract

**Background:**

Musculoskeletal pain and its risk factors are rarely assessed in studies on adolescent athletes. The aim was to identify risk factors at baseline that were associated with the persistence or development of musculoskeletal pain at a two-year follow-up in adolescent sport school students, and to study cross-sectional associations at follow-up between musculoskeletal pain and sports performance.

**Methods:**

Sport school students (79 boys and 52 girls, aged 14 years at baseline) were divided into infrequent (never–monthly) or frequent (weekly–almost daily) pain groups, based on frequency of pain using a pain mannequin. Logistic regression analyses were performed to study longitudinal associations between frequent pain at follow-up and baseline variables: pain group, number of regions with frequent pain, health status by EQ-5D, maturity offset (pre, average, or post peak height velocity), and sports (contact or non-contact). Linear regression analyses were used to study cross-sectional associations between pain groups and 20-m sprint, agility T-test, counter-movement jump, and grip strength at follow-up. Results were stratified by sex.

**Results:**

A higher percentage of girls than boys reported frequent pain at follow-up (62% vs. 37%; p = 0.005). In boys, frequent pain at follow-up was associated with being pre peak height velocity at baseline (OR 3.884, CI 1.146–13.171; p = 0.029) and participating in non-contact sports (OR 3.429, CI 1.001–11.748; p = 0.050). In girls, frequent pain at follow-up was associated with having frequent pain in two or more body regions at baseline (OR 3.600, CI 1.033–12.542; p = 0.044), having a worse health status at baseline (OR 3.571, CI 1.026–12.434; p = 0.045), and participating in non-contact sports (OR 8.282, CI 2.011–34.116; p = 0.003). In boys, frequent pain was associated with worse performances in 20-m sprint and counter-movement jump, but not in agility T-test and grip strength.

**Conclusions:**

Baseline risk factors for having frequent pain at follow-up were late maturation in boys, frequent pain and worse health status in girls, and participation in non-contact sports in both sexes. Boys with pain performed worse in sports tests. Coaches and school health-care services should pay attention to the risk factors and work towards preventing pain from becoming persistent.

## Background

Being physically active is associated with a better physical and mental health [1], but engaging in a high level of physical activity increases the risk of developing musculoskeletal pain [2, 3] and reporting of pain from multiple body sites [4]. In general, adolescents with pain have a worse self-reported health status [5, 6] based on questions concerning mobility, self-care, usual activities, pain, discomfort, anxiety, and depression. Pain may have a negative effect on the positive relationship between physical activity and health, and all factors should be assessed together.

Pain is a complex state defined as “An unpleasant sensory and emotional experience associated with, or resembling that associated with, actual or potential tissue damage” [7], which highlights the importance of also recognizing the subjective dimension of the condition. In sports, pain is often studied in relation to injuries, but according to the definition of pain, it does not necessarily have to be associated with having an injury [8]. Furthermore, pain in athletes requires special consideration since it may adversely affect health and sports performance [8, 9]. Recently, The International Olympic Committee (IOC) emphasized the need for assessing pain, and not only injuries, in athletes [9]. In light of this, there is a need to recognize risk factors for pain regardless of cause in adolescent athletes.

Musculoskeletal pain is common in children and adolescents, with a reported prevalence of current pain ranging between 4 and 60% [2, 10]. Girls generally have a higher prevalence of pain [2, 10, 11], and also of multi-site pain [5, 12], than boys. Different bio-psychosocial factors such as anxiety, reporting of a worse health status, severe sleeping problems, previous reporting of pain [13], and increasing age [14] may govern the persistence or development of pain. Pain in childhood is associated with reporting of pain in young adulthood [15], which is a consideration that should receive more attention regarding young athletes who experience persistent pain.

Pain prevalence increases as age increases, but the relationship between pain and maturity is unclear. The growth spurt, known as peak height velocity (PHV) and timing of maturity may differ significantly in adolescents of the same chronological age [16, 17], which is something that is usually not accounted for in sports. Sports are traditionally performed according to chronological age, and the heterogeneity in maturity timing may lead to poorly optimized training loads in certain individuals [18]. Despite findings indicating an increased risk of traumatic injuries at PHV and increased risk of overuse injuries after PHV [19], the overall relationship between maturity timing and injuries—and also that between maturity timing and pain—remain inconclusive [20].

Adolescent athletes may experience pain and still participate in sport, but few studies have investigated pain prevalence in this population. Moreover, pain is seldom studied together with assessment of health status, maturation, and sports performance. Due to a paucity in literature, it is unclear if pain is associated with a worse sports performance in adolescent athletes. The aim of this study was to identify risk factors at baseline that were associated with the persistence or development of musculoskeletal pain at a two-year follow-up in adolescent sport school students. A secondary aim was to study cross-sectional associations at follow-up between musculoskeletal pain and sports performance.

## Methods

Students who started the seventh grade in 2013, 2014, and 2015 (n = 233) at a Swedish sport school were invited to participate in this longitudinal study. The study is part of the research project “Malmö Youth Sport Study”, and baseline results have previously been published from a cross-sectional study [6]. The sport school is a public compulsory school which specializes in talent development and enrolls students after undergoing qualifying tests in their major sport. This concept is uncommon in Sweden since most sports are club-based and performed during leisure time. The sports at the school are football, ice hockey, basketball, and floorball (which are grouped as contact sports); and athletics, swimming, squash, badminton, tennis, figure skating, artistic gymnastics, and diving (which are grouped as non-contact sports). Sport-specific training is practiced during school hours (450 min per week) in addition to regular club practices and competitions.

Baseline data were collected at 13–14 years of age during the spring semester in the seventh grade. Follow-up data were collected at 15–16 years of age during the spring semester in the ninth grade. The participants completed questionnaires, anthropometric measures, and sports performance tests over 1–2 days (≤ 1 week apart) in school facilities. Experienced leaders collected the data and tests took place in random order. Before sports performance tests, an individual warm-up was performed (5–10 min). Sports performance tests were performed during similar conditions, in the same facility, and with the same equipment on both occasions.

The Regional Ethical Review Board in Lund, Sweden, approved the research project “Malmö Youth Sport Study” (Dnr 2012/745, Dnr 2014/270, and Dnr 2017/325). The study adhered to the ethical guidelines presented in the Declaration of Helsinki [21]. Written informed consent was obtained from both students and their guardians. The document also stated that participation was voluntary and that the students could withdraw from the study at any time without giving a reason. The study adhered to the Strengthening the Reporting of Observational Studies in Epidemiology (STROBE) guidelines [22].

### Outcome measures

#### Musculoskeletal pain

The experience of musculoskeletal pain (from here on referred to as pain) was self-reported using a pain mannequin and a numeric rating scale (NRS). Pain was, in accordance with the definition stated by the International Association for the Study of Pain [7], assessed as a subjective experience, focusing on localization and frequency of pain regardless of its origin. In line with this the students were instructed to indicate pain in the questionnaire if they were in pain, irrespective of cause. The pain mannequin [23] had 18 body regions, and for each region the participants specified the frequency of pain according to the following alternatives: never / rarely / monthly / weekly / more than once a week / almost daily) [2]. Content validity and adequate test–retest reliability have previously been demonstrated in adolescents for these pain frequency alternatives [24]. Pain intensity during the previous week was assessed on a NRS (0–10, signifying best to worst) [25].

#### Health status

The EQ-5D-3L (EQ-5D) assesses health status in the areas of mobility, self-care, usual activities, pain/discomfort, and anxiety/depression. The participant rated his/her experience of problems in each area using the response options: no problem, some problems, or extreme problems. An index (0–1.00, signifying worst health to best health) was calculated from responses [26].

#### Anthropometric measures

Height, sitting height, and body weight were measured to calculate maturity offset. Maturity offset describes the timing of physical maturation in relation to PHV. Zero indicates that the individual is at PHV, values above indicate years since PHV was reached, and values below indicate years left to PHV [27]. The method for measurements has been described in detail previously [6] and sex-specific equations were used for calculating maturity offset [27].

#### Sports performance tests

Twenty-meter sprint [28] and agility T-test were used to measure speed on an indoor track surface. The agility T-test protocol adhered to the one described by Pauole et al. [29], but included cones. Infrared single-beam timing gates (MuscleLab; Ergotest Innovation, Porsgrunn, Norway) measured time, and the starting line was placed 0.5 m behind the timing gates. The 20-m sprint was performed three times, and the agility T-test was performed twice. The fastest time (to the nearest 0.01 s) was used in analyses.

Counter-movement jump with arm swing (CMJ-AS) [30] performed on an infrared mat (MuscleLab; Ergotest Innovation) was used to measure maximal muscular power in the lower extremities. The highest jump (registered to the nearest 0.1 cm) out of three jumps was used in analyses.

Maximal grip strength was measured with a hand grip dynamometer (KERN Sohn GmbH, Balingen, Germany). The test adhered to the Southampton protocol, but with forearm resting on a table. Three consecutive attempts were measured for each hand. The best result (registered to the nearest 0.1 kg), independent of hand, was used in analyses [31].

### Statistical analysis

At baseline and at follow-up, the students were grouped as having infrequent pain (never / rarely / monthly) or as having frequent pain (weekly / more than once a week / almost daily) according to the highest reported pain frequency in any body region. The number of regions with frequent pain was also calculated and grouped as 0, 1, and ≥ 2. EQ-5D was dichotomized into the best tertiary (index 1.00) vs. the two worst tertiaries (index < 1.00). Maturity offset was categorized into pre (< − 1.0 year), average (± 1.0 year), and post (> 1.0 year) PHV. Sports were categorized into contact sports and non-contact sports.

Data are presented as mean and standard deviation (SD) or as number and percentage. Results are mainly presented and analyzed separately for boys and girls due to sex-specific differences in associations between pain, maturity offset, and sports performance. Selected comparisons between boys and girls were analyzed with chi^2^-tests or with logistic regression analysis. Logistic regression analyses were performed to study risk factors for frequent pain. Independent baseline variables (pain groups, number of regions with frequent pain, pain intensity, EQ-5D categories, maturity offset categories, and sports categories) were inserted separately into analyses with frequent pain at follow-up as dependent variable. Results are presented as odds ratio (OR) with 95% confidence interval (CI). Linear regression analysis was used to study cross-sectional associations between frequent pain at follow-up and sports performance tests (20-m sprint, agility T-test, counter-movement jump, and grip strength) at follow-up. Variables were inserted separately into analyses. Results are presented as beta coefficient (B) and 95% CI. Statistical significance was set to p-values ≤ 0.05 and analyses were done using IBM SPSS Statistics software v.24.0 (IBM Corp., Armonk, NY, USA).

## Results

**Fig. 1:**
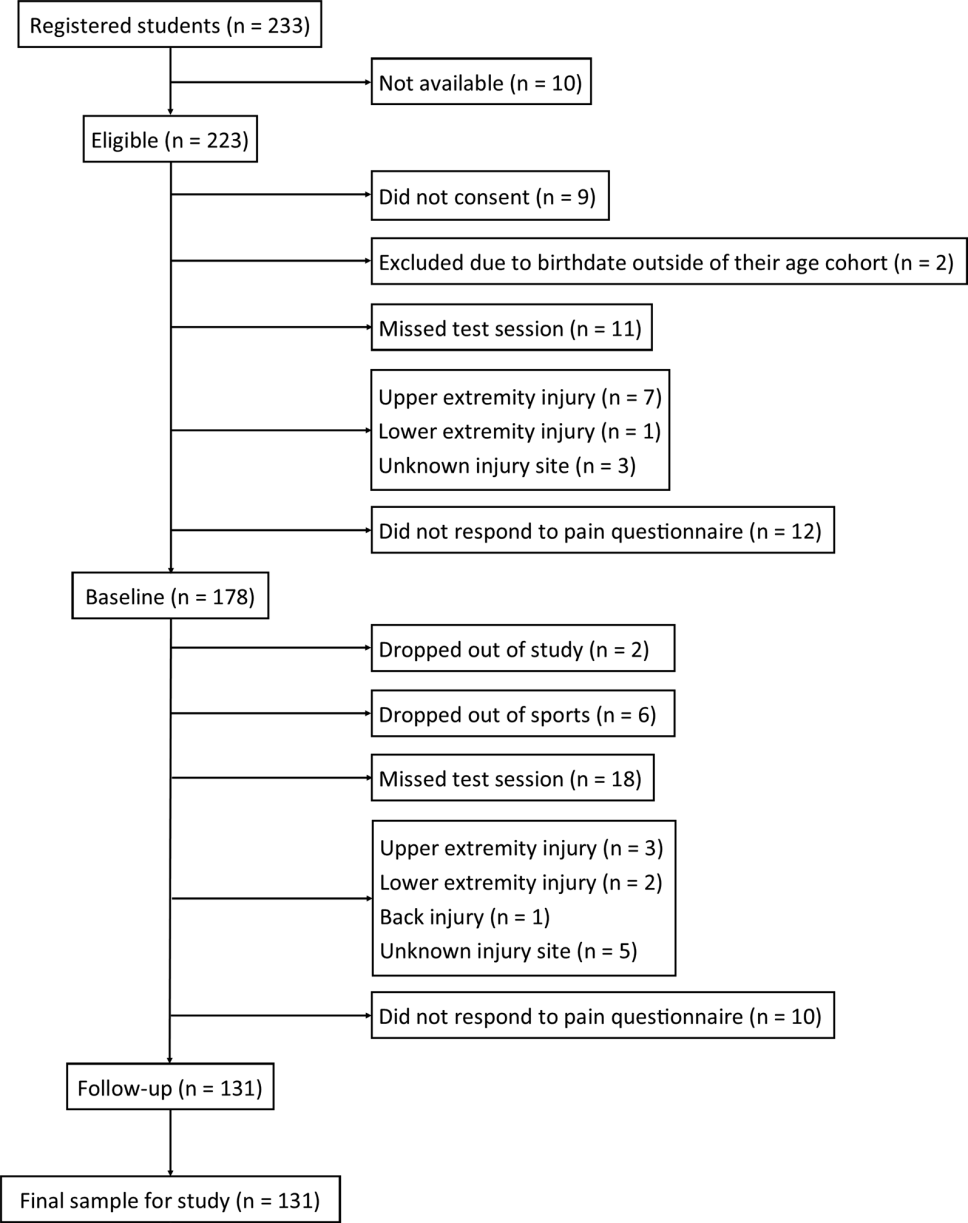
Flow chart

Of 233 registered students, 223 students were eligible to participate in the study. Nine students did not consent, leaving 214 students. Students were excluded at baseline if they were born outside of their ordinary age cohort, missed the test sessions, reported injuries, or did not respond to the pain questionnaire (n = 36), giving 178 students at baseline. Two students dropped out of the study from baseline to follow-up. Students were excluded at follow-up if they dropped out from their major sport, missed the test sessions, reported injuries, or did not respond to the pain questionnaire (n = 45), resulting in a final sample of 131 students (79 boys, 60%; and 52 girls, 40%; mean age 13.98 ± SD 0.26 years at baseline and 16.00 ± SD 0.26 years at follow-up) included in the analyses (Fig. 1). The 47 students who were lost to follow-up (27 boys, 57%; and 20 girls, 43%) did not differ significantly in terms of pain group distribution, mean value for EQ-5D, or in sports categorization at baseline from the final sample.

Descriptive data for baseline and follow-up variables are presented in Tables 1 and 2. In total, 54% (n = 71) of the students reported having frequent pain at baseline, and 47% (n = 61) at follow-up. A higher percentage of girls than boys were categorized into the frequent pain group at follow-up (Table 2; p = 0.005). The most commonly reported pain regions at baseline were the knees (27%) and the lower legs/feet (17%), and at follow-up the lower back (21%) and the knees (18%).

**Table 1:** Descriptive data for boys and girls at baseline (n = 131)

	Baseline			
	Boys (n = 79)		Girls (n = 52)	
	n	Mean ± SD or %	n	Mean ± SD or %
Pain group^a^				
Infrequent pain group	38	48%	22	42%
Frequent pain group	41	52%	30	58%
Number of regions with frequent pain				
1 region	9	11%	6	12%
≥ 2 regions^b^	32	41%	24	46%
Pain intensity, NRS 0–10^c^	76	3.3 ± 2.5	49	2.9 ± 2.0
EQ-5D (0.00–1.00)^d^	77	0.86 ± 0.14	50	0.84 ± 0.14
1.00	30	39%	16	32%
< 1.00	47	61%	34	68%
Height (cm)	79	166.2 ± 9.5	51	163.9 ± 5.6
Body weight (kg)	79	53.4 ± 9.8	52	54.6 ± 6.6
Chronological age (years)	79	13.96 ± 0.26	52	13.99 ± 0.25
Maturity offset (years)	79	–0.28 ± 0.76	51	1.73 ± 0.42
Pre PHV (< − 1.0 year)	14	18%	0	0%
Average PHV (± 1.0 year)	60	76%	1	2%
Post PHV (> 1.0 year)	5	6%	50	98%
20-m sprint (s)	70	3.34 ± 0.20	48	3.40 ± 0.16
Agility T-test (s)	68	10.62 ± 0.60	48	10.98 ± 0.56
CMJ-AS (cm)	69	34.9 ± 5.9	47	33.0 ± 4.8
Grip strength (kg)	78	30.3 ± 7.3	52	28.2 ± 4.2
Sports category^e^				
Contact sports	66	84%	30	58%
Non-contact sports	13	16%	22	42%

The results are presented as mean ± SD or as n and %. Pain group and sports category were analyzed with chi^2^ tests

NRS, numeric rating scale; PHV, peak height velocity; CMJ-AS, counter-movement jump with arm swing

^a^Analyzed with chi^2^ test. Boys vs. girls, p = 0.515

^b^Range for boys were 2–8 regions and for girls 2–7 regions

^c^Scored from best to worst

^d^Scored from worst to best. EQ-5D was dichotomized according to the best tertiary (1.00) vs. the two worst tertiaries (< 1.00)

^e^Analyzed with chi^2^ test. Boys vs. girls, p = 0.001

**Table 2:** Descriptive data for boys and girls at follow-up (n = 131)

	Follow-up			
	Boys (n = 79)		Girls (n = 52)	
	n	Mean ± SD or %	n	Mean ± SD or %
Pain group^a^				
Infrequent pain group	50	63%	20	38%
Frequent pain group	29	37%	32	62%
Number of regions with frequent pain				
1 region	13	17%	17	33%
≥ 2 regions^b^	16	20%	15	29%
Pain intensity, NRS 0–10^c^	78	2.7 ± 2.5	50	3.2 ± 2.4
EQ-5D (0.00–1.00)^d^	78	0.87 ± 0.17	52	0.85 ± 0.14
1.00	35	45%	20	38%
< 1.00	43	55%	32	62%
Height (cm)	75	177.7 ± 6.8	46	166.9 ± 5.3
Body weight (kg)	75	66.6 ± 8.9	46	60.5 ± 6.2
Chronological age (years)	79	15.99 ± 0.26	52	16.02 ± 0.26
Maturity offset (years)	74	1.82 ± 0.71	45	3.09 ± 0.38
Pre PHV (< − 1.0 year)	0	0%	0	0%
Average PHV (± 1.0 year)	11	15%	0	0%
Post PHV (> 1.0 year)	63	85%	45	100%
20-m sprint (s)	62	3.14 ± 0.19	40	3.42 ± 0.18
Agility T-test (s)	59	10.13 ± 0.52	38	10.81 ± 0.65
CMJ-AS (cm)	60	42.3 ± 7.2	43	32.8 ± 5.7
Grip strength (kg)	75	40.7 ± 6.5	46	30.3 ± 4.0

The results are presented as mean ± SD or as n and %. Pain group was analyzed with chi^2^ tests

NRS, numeric rating scale; PHV, peak height velocity; CMJ-AS, counter-movement jump with arm swing

^a^Analyzed with chi^2^ test. Boys vs. girls, p = 0.005

^b^Range for boys were 2–7 regions and for girls 2–12 regions

^c^Scored from best to worst

^d^Scored from worst to best. EQ-5D was dichotomized according to the best tertiary (1.00) vs. the two worst tertiaries (< 1.00)

### Baseline predictors of frequent pain at follow-up

Overall, girls had a higher risk than boys for belonging to the frequent pain group at follow-up (OR 2.759, 95% CI 1.340–5.679; p = 0.006). When the results were stratified by sex, being pre PHV and participating in non-contact sports were found to be risk factors in boys for having frequent pain at follow-up. In girls, frequent pain, pain from two or more body regions, an index below 1.00 in EQ-5D, and participation in non-contact sports were risk factors for having frequent pain at follow-up. Maturity offset categories could not be analyzed in girls because the majority of girls were classified as being post PHV at baseline (Table 3).

**Table 3:** Associations between baseline variables and frequent pain at follow-up for boys and girls

Baseline variables	Boys (n = 79)		Girls (n = 52)	
	Frequent pain at follow-up		Frequent pain at follow-up	
	OR	(95% CI; p-value)	OR	(95% CI; p-value)
Pain group				
Infrequent (ref)	1.000		1.000	
Frequent	2.418	(0.937–6.238; p = 0.068)	3.300	(1.029–10.588; p = 0.045)
Number of regions with frequent pain				
0 (ref)	1.000		1.000	
1	2.240	(0.500–10.039; p = 0.292)	2.400	(0.361–15.942; p = 0.365)
≥ 2	2.471	(0.907–6.727; p = 0.077)	3.600	(1.033–12.542; p = 0.044)
Pain intensity, NRS 0–10^a^	1.087	(0.902–1.310; p = 0.382)	1.335	(0.961–1.855; p = 0.085)
EQ-5D^b^				
1.00 (ref)	1.000		1.000	
< 1.00	1.728	(0.654–4.567; p = 0.270)	3.571	(1.026–12.434; p = 0.045)
Maturity offset (years)	0.530	(0.272–1.032; p = 0.062)	1.301	(0.328–5.156; p = 0.708)
Maturity offset category				
Average PHV (± 1.0 year; ref)	1.000			
Post PHV (> 1.0 year)	0.539	(0.056–5.159; p = 0.592)		^c^
Pre PHV (< − 1.0 year)	3.884	(1.146–13.171; p = 0.029)		
Sports category				
Contact sports (ref)	1.000		1.000	
Non-contact sports	3.429	(1.001–11.748; p = 0.050)	8.282	(2.011–34.116; p = 0.003)

Results were analyzed with logistic regression analysis and are presented as odds ratio (OR) and 95% confidence interval (CI). All variables were inserted separately in analyses

NRS, numeric rating scale; PHV, peak height velocity; Ref, reference category

^a^Scored from best to worst

^b^EQ-5D was dichotomized according to the best tertiary (1.00) vs. the two worst tertiaries (< 1.00)

^c^Maturity offset categories could not be analyzed in girls because the majority of girls were classified as being post PHV at baseline

### Associations between frequent pain and sports performance at follow-up

The cross-sectional associations between frequent pain at follow-up and sports performance at follow-up was analyzed. Boys with frequent pain at follow-up performed worse in 20-m sprint and in CMJ-AS at follow-up than boys in the infrequent pain group, whereas pain group belonging did not influence the results of sports performance tests in girls at follow-up (Table 4).

**Table 4:** Associations between frequent pain at follow-up and sports performance at follow-up in boys and girls

Follow-up	Follow-up							
	20-m sprint (s)		Agility T-test (s)		CMJ-AS (cm)		Grip strength (kg)	
	B	(95% CI; p-value)	B	(95% CI; p-value)	B	(95% CI; p-value)	B	(95% CI; p-value)
Pain group boys								
Infrequent (ref)	0.00		0.00		0.0		0.0	
Frequent	0.12	(0.03 to 0.22; p = 0.011)	0.20	(− 0.07 to 0.48; p = 0.146)	− 4.0	(− 7.7 to − 0.2; p = 0.041)	− 2.2	(− 5.3 to 0.9; p = 0.159)
Pain group girls								
Infrequent (ref)	0.00		0.00		0.0		0.0	
Frequent	0.01	(− 0.11 to 0.13; p = 0.858)	0.04	(− 0.41 to 0.49; p = 0.860)	0.9	(− 2.9 to 4.6; p = 0.643)	1.0	(− 1.4 to 3.5; p = 0.399)

Results were analyzed with linear regression analysis and presented as beta coefficient (B) and 95% confidence interval (CI). All variables were inserted separately in analyses

CMJ-AS, counter-movement jump with arm swing; Ref, reference category

## Discussion

In this longitudinal study on sport school students, one in every two students reported having frequent pain at baseline or at the two-year follow-up. Girls were at higher risk of having frequent pain at follow-up than boys. Being pre PHV at baseline was a risk factor for boys, whereas having frequent pain in two or more body regions and reporting a worse health status at baseline were risk factors for girls for belonging to the frequent pain group two years later. Participation in non-contact sports was a risk factor for having frequent pain at follow-up in both boys and girls. Having frequent pain at follow-up was associated with a worse sports performance at follow-up in boys, but no associations between frequent pain and sports performance were found in girls.

The overall prevalence of pain in this study was similar to that reported in population-based studies [2, 10, 32]. Still, the finding that one in two adolescent athletes experienced frequent pain in our study is concerning, since physical activity from a public health point of view would be expected to lead to better health and less pain. Legault et al. [33] investigated the 6-month prevalence of pain in adolescent athletes and found a prevalence of 38%. The methodology used for measuring pain differs between studies with regard to frequency, duration, intensity, and localization, which probably contributes to the variation in pain prevalence reported.

The prevalence of frequent pain at follow-up was higher in girls than in boys in our study. Previous research has shown that girls are at higher risk of experiencing pain than boys [2, 10], which was reflected by our athletic sample as well. The over-representation of girls with pain remains to be explained, but it is possibly a combination of biological, psychological, and social considerations that governs the experiencing and persistence of pain in boys and girls.

Frequent pain at baseline was a risk factor for frequent pain at follow-up for girls in this study, but the number of regions with frequent pain was also of importance. Pain in two or more body regions was associated with having frequent pain two years later for girls. The same was not found for girls reporting pain in only one body region. For boys there was a similar trend as that seen in girls. A previous cohort study found high persistence rates of multisite pain over time in both boys and girls [12], and it is possible that a larger sample of boys in our study could have strengthen the analysis. Rathleff et al. [2] reported a prevalence of 30% for multisite pain, which is slightly lower than the prevalence of 40–46% seen in our study on adolescent athletes.

Having a worse health status at baseline was a risk factor for having frequent pain at follow-up in this study, which was an association that was only seen in girls. The association between pain and poorer health status is supported by the results of a population-based study on pain in adolescents [5]. Since the present study included both persistence and development of frequent pain over time, it is not possible to say whether or not a worse health status at baseline is predictive of pain in the future. Psychological symptoms in children and adolescents appear to be predictive of reporting musculoskeletal complaints two years later [34], which emphasizes the need for monitoring of health status and pain together more closely in adolescent athletes. The pressure to perform in sports may lead to pain, and pain that is not properly managed over time may lead to poorer health. Both athletes and their surrounding teams must recognize that pain management requires a bio-psychosocial approach [8]. Sustainability in the health of adolescent athletes is an important goal for youth sports [35].

In comparison with boys from the average and post PHV group, boys in the pre PHV group were at higher risk of being in the frequent pain group at follow-up. Interestingly, in a review on pain and maturity, Swain et al. [20] stated that there was not enough evidence to conclude that pain increases as maturity advances in general populations. Our finding of late maturing boys still reporting having pain after two years adds to the ongoing discussion about the relationship between pain and maturity, but it is uncertain whether our results are specific to late maturing boys enrolled in a competitive sports school or if late maturity per se might increase the risk for pain among adolescent boys in general. Further studies on both adolescent athletes and boys in general are required to improve our knowledge in this area.

In the present study, the students who participated in non-contact sports were more likely to report having frequent pain at the follow-up. A previous population-based study on adolescents [32] demonstrated that the risk of having frequent pain in specific regions varied depending on type of sport and sex. Boys in team sports, such as football and handball, had a lower risk of having neck and shoulder pain, but a higher risk of having lower extremity pain in comparison with non-active boys. This result was not seen in girls from team sports. Girls in endurance sports, such as cross-country skiing and running, had a lower risk of having neck and shoulder pain and low back pain than non-active girls, but for boys there were no differences in risk of pain between boys in endurance sports and non-active boys [32]. The type of sport and sex appears to be important when studying frequency and localization of pain and should be considered in future studies.

Pain and type of sport can also be discussed in relation to injuries. Overuse injuries appear to be more common in non-contact sports than in contact sports [36], which could be in line with the increased risk of pain seen in non-contact sport athletes in our study. Contact sports appear to be associated with a high risk of traumatic injuries [36]. Traumatic injuries usually include time lost from participation, and in this study the injured students were excluded mostly due to the fact that they could not participate in sports tests. The reported pain in this study is thus less likely to be due to current acute injuries. Hainline et al. [9] emphasized both pain management (the bio-psychosocial perspective) and injury management (focusing on the musculoskeletal state) for athletes, since pain may persist independently of injury—or after the tissue has healed. Since we studied the experience of pain regardless of cause, the results should be interpreted from this standpoint.

Frequent pain was found to be associated with a worse sports performance in the 20-m sprint and CMJ-AS at follow-up in boys. The same analysis (but controlled for maturity offset) was previously done for baseline, which showed similar results [6]. Since maturity offset is the most accurate around average PHV [16, 17], it was omitted from analysis at follow-up. Due to heterogeneity in the type of sports represented at the school and small differences in sports performance between pain groups, it is difficult to evaluate whether the negative effect of frequent pain on sports performance has any practical implications. Future studies of pain in athletic samples should assess functional ability in addition to questions about pain.

The limitations of this study should also be discussed. The drop-out rate from baseline to follow-up was 26%, but the students who were lost to follow-up did not differ significantly from the sample included regarding baseline variables. The study had to rely on the limited number of participants that could be included from this unique sport school, which may invoke a power problem in analyses with non-significant, although interesting, results. As an example, a post-hoc analysis with the observed absolute difference of 25% for the outcome of frequent pain in boys at follow-up, gave a statistical power of 60% based on two-tailed test with the statistical significance set to p-value < 0.05. Having a larger sample size might have given higher power in logistic regressions (where non-significant trends were identified in boys) and might have allowed multiple regression analyses, which could not be performed with the sample size used.

Adolescents admitted to the sports school were already pre-selected because of their sports talent, which could make it difficult to apply the results to a more general population [37]. It is also a limitation that time spent or intensity of training in regular club practices and competitions outside of school hours was not collected. This may vary depending on type and level of sports. The selected sports performance tests are commonly used when evaluating physical capacity in adolescents, but sports performance is a broader concept involving technical and tactical elements, which was not assessed in the present study.

Almost all the girls in this sport school study were classified as post PHV. It would have been interesting to determine how timing in physical maturity affected pain in girls, but since they were 13–14 years of age during their first year at the sport school (baseline), and girls generally reach PHV at the age of 12 [17], the study started too late to capture PHV in girls. Analyzing maturity offset as a continuous variable gave no further information.

To estimate maturity by anthropometric measurements and sex-specific equations in adolescents is an uncertain method in comparison with skeletal age assessments [38], but it is one of few non-invasive methods available. An update of maturity offset equations was published a few years ago [39], but was not used in the previously published cross-sectional study [6] or in the present longitudinal study. Analyses with the updated equations (data not shown) did not change the results from or the conclusions made in the present manuscript. Future studies in the field should take the various limitations with maturity estimation into consideration.

The study had several strengths that are worth highlighting. Firstly, the longitudinal study design enabled us to study risk factors for persistence or development of frequent pain over two years. Secondly, the study involved both boys and girls attending the same sport school, which proved to be valuable in terms of increasing our knowledge of sex-specific differences in risk factors for pain in adolescent athletes. Thirdly, a variety of sports were represented, which strengthened the applicability of the results.

Pain in childhood is associated with reporting of pain in young adulthood, and persistent pain has negative effects on physical, psychological, and social wellbeing. In the IOC consensus statement on pain management in athletes, it is emphasized that pain should be understood as a condition that is influenced by bio-psychosocial factors [9]. The results from the present study are in line with the IOCs recommendations, and give scientific support for parents and for the support staff surrounding the adolescent athlete in being observant of the increased risk of developing persistent pain. The findings can also assist in developing pain preventive interventions in adolescent athletes who are aiming for a future professional career. It is of utmost importance to recognize students who are at higher risk of developing pain early on, to prevent pain from becoming persistent.

## Conclusions

Frequent pain is common in sport school students, especially in girls. Baseline risk factors identified were late physical maturation in boys, frequent pain and a worse health status in girls, and participation in non-contact sports in both sexes. Frequent pain at follow-up was associated with worse sports performance in boys, but not in girls. Parents, coaches, teachers, and school health-care services should pay attention to the risk factors for pain in young athletes and work towards preventing pain from becoming persistent.

## Data Availability

The datasets generated and/or analyzed during the current study are not publicly available due to ethical reasons.

## Abbreviations

B: Beta coefficient
CI: Confidence interval
CMJ-AS: Countermovement jump with arm swing
IOC: International Olympic Committee
NRS: Numeric rating scale
OR: Odds ratio
PHV: Peak height velocity
Ref: Reference category
SD: Standard deviation
